# Probing aqueous ions with non-local Auger relaxation†

**DOI:** 10.1039/d2cp00227b

**Published:** 2022-03-23

**Authors:** Geethanjali Gopakumar, Eva Muchová, Isaak Unger, Sebastian Malerz, Florian Trinter, Gunnar Öhrwall, Filippo Lipparini, Benedetta Mennucci, Denis Céolin, Carl Caleman, Iain Wilkinson, Bernd Winter, Petr Slavíček, Uwe Hergenhahn, Olle Björneholm

**Affiliations:** Department of Physics and Astronomy, Uppsala University Box 516 SE-751 20 Uppsala Sweden geethanjali.gopakumar@physics.uu.se; Department of Physical Chemistry, University of Chemistry and Technology Technická 5 Prague 6 166 28 Czech Republic petr.slavicek@vscht.cz; Center for Free-Electron Laser Science, DESY Notkestraße 85 22607 Hamburg Germany; Molecular Physics Department, Fritz-Haber-Institut der Max-Planck-Gesellschaft Faradayweg 4-6 14195 Berlin Germany hergenhahn@fhi-berlin.mpg.de; Institut für Kernphysik, Goethe-Universität Frankfurt am Main Max-von-Laue-Straße 1 60438 Frankfurt am Main Germany; MAX IV Laboratory, Lund University Box 118 SE-22100 Lund Sweden; Department of Chemistry and Industrial Chemistry, University of Pisa Via Giuseppe Moruzzi 13 56124 Pisa Italy; Synchrotron SOLEIL, L'Orme des Merisiers, Saint-Aubin BP 48, 91192 Gif-sur-Yvette Cedex Paris France; Department of Locally-Sensitive & Time-Resolved Spectroscopy Helmholtz-Zentrum Berlin für Materialien und Energie 14109 Berlin Germany

## Abstract

Non-local analogues of Auger decay are increasingly recognized as important relaxation processes in the condensed phase. Here, we explore non-local autoionization, specifically Intermolecular Coulombic Decay (ICD), of a series of aqueous-phase isoelectronic cations following 1s core-level ionization. In particular, we focus on Na^+^, Mg^2+^, and Al^3+^ ions. We unambiguously identify the ICD contribution to the K-edge Auger spectrum. The different strength of the ion–water interactions is manifested by varying intensities of the respective signals: the ICD signal intensity is greatest for the Al^3+^ case, weaker for Mg^2+^, and absent for weakly-solvent-bound Na^+^. With the assistance of *ab initio* calculations and molecular dynamics simulations, we provide a microscopic understanding of the non-local decay processes. We assign the ICD signals to decay processes ending in two-hole states, delocalized between the central ion and neighbouring water. Importantly, these processes are shown to be highly selective with respect to the promoted water solvent ionization channels. Furthermore, using a core-hole-clock analysis, the associated ICD timescales are estimated to be around 76 fs for Mg^2+^ and 34 fs for Al^3+^. Building on these results, we argue that Auger and ICD spectroscopy represents a unique tool for the exploration of intra- and inter-molecular structure in the liquid phase, simultaneously providing both structural and electronic information.

## Abstract 2

Non-local analogues of Auger decay, such as Intermolecular Coulombic Decay, are increasingly recognized as important relaxation processes in aqueous electrolytes.

## Graphical abstract

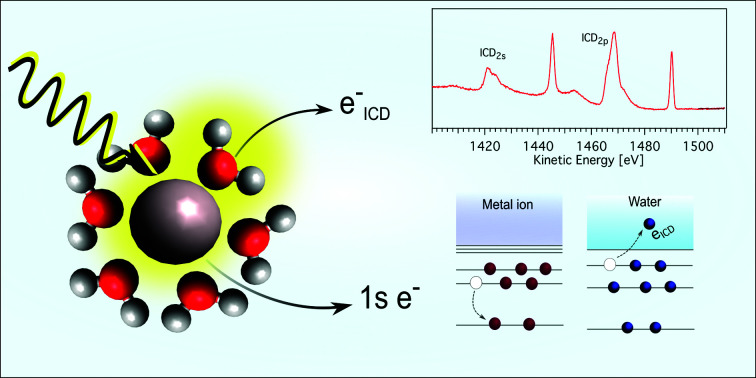

## 1 Introduction

The recent exploration of the molecular structure in soft condensed matter and liquids forced a revision of a number of previously helpful paradigms and revealed novel, hitherto unobserved effects.^1–3^ In the context of the interaction of radiation and liquids, the discovery of Intermolecular Coulombic Decay^4–6^ (ICD) is probably one of the most exciting phenomena. Upon first glance, ICD shows similarities with the well-known Auger decay, however, it is a decay mechanism characteristic of condensed systems only. It takes place in an atom or molecule after an inner-valence or inner-shell vacancy is created. The vacancy is refilled with a valence electron and the released energy ionizes a neighbouring atom or molecule. The whole process leads to an energetically favourable delocalized two-hole state {Center^+^⋯Surrounding^+^} in contrast to an Auger decay, where a higher energy {Center^2+^} state is formed.

ICD and other non-local Auger-type processes have entered the field of X-ray science relatively recently and have primarily been discussed in the context of their role in radiation chemistry or X-ray photochemistry.^7–9^ The simultaneous ionization of neighbouring species leads to completely new reaction channels that should be considered within the overall radiolysis mechanisms.^10^ Non-local Auger phenomena also offer new possibilities to probe liquid-phase molecular structure, as the decay processes are highly dependent on the intermolecular distances. Some of us have already suggested that the analysis of electron spectra after autoionization of an ionic metal centre could be interpreted with respect to the environment of the respective ion.^11,12^ Moreover, in the liquid state, ICD spectra of metals involving surrounding water molecules have already been observed.^6,13,14^ In fact, the sensitivity of electronic relaxation spectra to the chemical environment of the emitter started to be discussed immediately after the discovery of ICD.^15,16^ Much like Förster energy transfer, ICD also exhibits a 1/*R*^6^ dependence on the intermolecular distance, *R* (ref. 6). The impact of ICD, however, is much broader than Förster transfer, since every atom or molecule may act as the receiving end of an inter-centre energy transfer, leading to the ejection of a secondary electron. Corresponding X-ray spectroscopies thus represent a relatively new tool to reveal liquid structure, complementing more conventional techniques such as dielectric spectroscopy or neutron scattering. Previously, the latter methods have been used in conjunction with molecular dynamics (MD) simulations to infer the arrangement of anions and cations in an electrolyte solution.^17^ X-ray-based spectroscopies and associated theoretical treatments represent an alternative to these well-established techniques due to their ability to directly and atom-specifically probe the electronic structure of the sample, while simultaneously delivering comparable information about the geometrical microstructure. With the combination of bright high-energy light sources, accurate electron analyzers, and liquid-microjet approaches, we can now relate the liquid structure to its signature in electron spectroscopy.^18^ Hand-in-hand with advances in X-ray-based experimental techniques for probing liquids' electronic structure, also the methods to simulate such structures have advanced immensely.^19–23^

Despite the enormous interest in the non-local Auger-like decay processes in the last decade, their application to elucidate liquid-phase molecular structure remains scarce. In the cases probed so far, these non-local signatures were weak, and since LVV Auger decay was considered, they consisted of a convolution of the water and metal valence shells.^13,24^ This complicated the interpretation of the spectral signatures. In the present work, we focus on the ICD signatures in the K-shell spectra. Such measurements provide a clear-cut case: the signal should be dominated by a valence vacancy of the surrounding and an L-shell vacancy in the metal centre, with a fairly well-defined energy.

We present experimental K-shell Auger and ICD spectra of aqueous-phase Al, Mg, and Na ions, associated with AlCl_3_, MgCl_2_, and NaCl solutions. These cations were selected because they are isoelectronic but represent different types of interaction with neighbouring molecules. While the sodium cation is only weakly bound to neighbouring water molecules, dicationic magnesium is more strongly coordinated and the aluminium ion forms a regular coordination-covalent bond; the ICD signal is shown to reflect this diversity. The measurements are accompanied by molecular dynamics (MD) simulations and *ab initio* calculations, which aid in the interpretation of the experimental data and allow for an in-depth analysis of the spectra.

## 2 Methods

### 2.1 Experimental methods

The Auger and ICD electron spectra were measured using the EASI photoemission setup,^25^ equipped with a liquid microjet and a hemispherical electron energy analyzer, at the P04 beamline of the synchrotron radiation facility PETRA III, DESY, Hamburg.^26^ The beamline has a high on-target photon flux of about 2 × 10^12^ photons s^−1^ at a resolving power of 10 000, with a photon-energy tuning range spanning 250–3000 eV, and variable circular polarization.^27^ The beamline's 1200 lines per mm grating yields a photon-energy resolution of 250 meV at 1200 eV photon energy and 350 meV at 1500 eV, using an exit-slit opening of 100 μm. At these settings, the vertical spot size amounts to approximately 35 μm. The general properties of the liquid-microjet system are described elsewhere.^25,28^ The liquid microjet of the sample solutions was introduced into the vacuum chamber using an HPLC pump at a flow rate of 0.8 ml min^−1^ with a backing pressure of ≤ 12 bar, and was directed horizontally. The glass capillary nozzle used to introduce the sample into the chamber had an inner diameter of 28 μm. The synchrotron radiation was incident perpendicular to the flow of the solution. A near-ambient-pressure hemispherical electron analyzer (Scienta Omicron HiPP-3), mounted at a 50° backward-scattering angle with respect to the beamline (near magic angle), was used to measure the electron kinetic energy.^25^ The solutions were prepared by dissolving commercially purchased AlCl_3_, MgCl_2_, and NaCl (Sigma-Aldrich with purity > 98%) salts in MilliQ (18.2 MΩ cm^−1^) water. Aqueous solutions of MgCl_2_ and NaCl had a concentration of 1 M, while that for AlCl_3_ was 2 M. At pH ≤ 4, the aluminium is predominantly present as the aluminium hexahydrate cation ([Al(H_2_O)_6_]^3+^), *i.e.*, the Al^3+^ surrounded by six water molecules.^29^ The utilised aluminium chloride solutions were highly acidic (pH < 2), which results in the exclusion of large amounts of chloride from direct contact with Al^3+^.

The kinetic energy of the electrons produced in the decay processes, both local (Auger) and non-local (ICD), are independent of photon energy (*hν*). Therefore, to distinguish the decay features from the photoelectron peaks, electron spectra were measured for two photon energies differing by 3 eV in all cases. Hemispherical-electron-analyzer pass energies of 100 or 200 eV were used together with an analyzer slit width of 800 μm, leading to an estimated analyzer resolution between 0.2 and 0.4 eV.

Photon energies and kinetic energies were calibrated as detailed in the ESI,† in part making use of additional measurements carried out at the U49-2_PGM-1 beamline of the BESSY II synchrotron-radiation source at the Helmholtz–Zentrum Berlin für Materialien und Energie, using the SOL^3^PES setup for liquid-jet photoemission spectroscopy.^28,30^

The observed ICD features were interpreted by a line-shape analysis using Voigt profiles in the SPANCF (Spectrum Analysis by Curve Fitting) macro package^31^ for Igor Pro (Wavemetrics, Inc., Lake Oswego, USA). In our case, either a 2p or 2s electron of the cation recombines with the 1s hole following direct photoemission, and the released energy leads to the secondary emission of electrons from the water valence states. The multipeak structure of each individual ICD feature (see Fig. 2) thus arises from the release of electrons from different valence states. During the data fitting process, the kinetic energy, the intensity of the peaks, and the Gaussian width were free to vary and the Lorentzian width was considered to be that of the cation 2p orbital (see Fig. 4). The relative kinetic energy differences of these peaks are almost the same as the relative binding energies of the water valence-band peaks.^32^ The main KLL Auger peak notably has a higher-kinetic-energy asymmetric tail that could be modelled by a PCI (post-collision interaction) profile, as originally developed for gas-phase work.

**Fig. 1:**
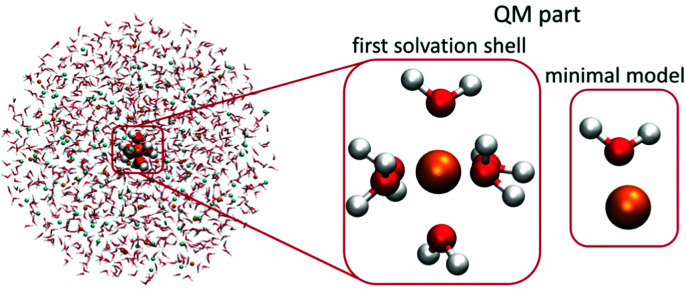
Scheme of the two QM/MMPol models employed in the present study. The QM part contained one metal cation and six water molecules or one cation and one water molecule (minimal model), the remainder of the 20 Å-radius sphere was treated at the polarizable embedding MM level.

**Fig. 2:**
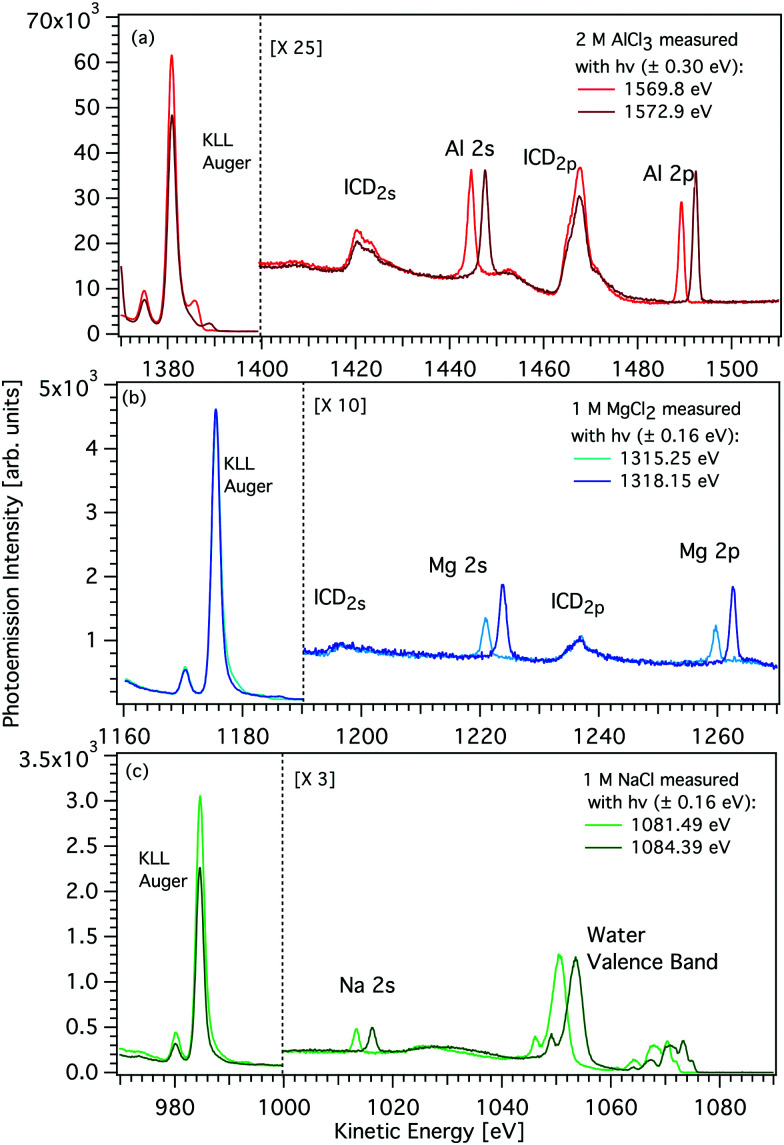
Photoemission spectra of aqueous solutions of AlCl_3_, MgCl_2_, and NaCl (panels a–c). Two photon energies were selected and implemented slightly above the respective K-edge of the metal and the resulting spectra are compared. In each panel, the left-hand side shows the main (KLL) Auger peak, and the right-hand side photoemission peaks due to direct L-shell ionization and due to K-shell ICD involving an L-shell electron (see labels). In the case of Na, no ICD peak can be observed, and the water valence band extends into the observed spectral region. Right-hand side spectra were scaled to improve visibility. See text for details.

**Fig. 3:**
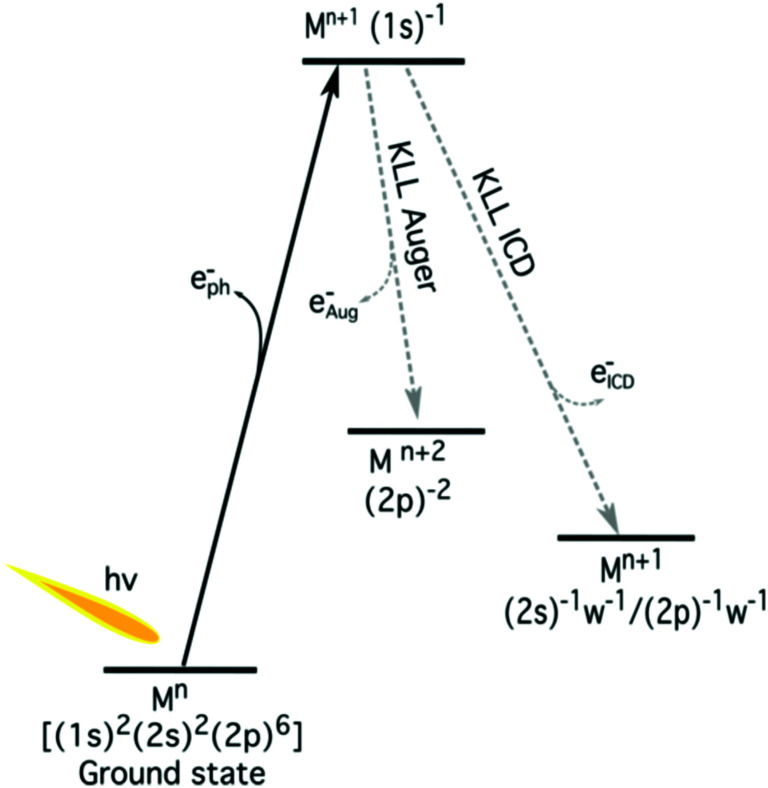
The schematic energy-level diagram of the relaxation of a 1s core hole in aqueous metal (M) ions. The local decay channel (KLL Auger decay) and non-local decay (ICD) are shown. The neighbouring water molecules are indicated by ‘w’.

**Fig. 4:**
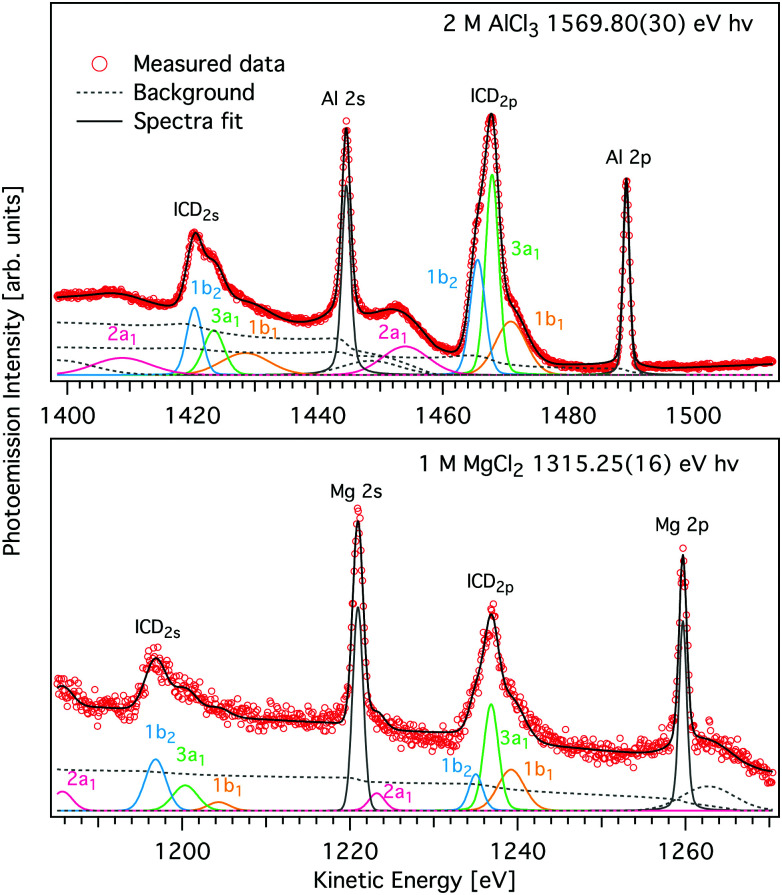
Magnified view of the L-shell photoelectron and ICD peaks of AlCl_3_ (upper panel) and MgCl_2_ (lower panel) aqueous solutions shown in Fig. 2 (symbols). Spectra were measured with *hν* = 1569.8 eV and 1315.25 eV, respectively. A decomposition of the ICD features into components pertaining to final states of different energy, modelled by Voigt profiles and found by least squares curve fitting is shown by coloured traces (see text for details). Subcomponents of the ICD structure correspond to different water valence-band orbitals, namely 1b_1_ (yellow), 3a_1_ (green), 1b_2_ (blue), and 2a_1_ (purple) and appear for both ICD_2p_ and ICD_2s_. The peak fits to the two spectra measured with higher *hν* are given in Fig. S2 of the ESI.†

### 2.2 Theoretical methods

#### Molecular dynamics

Classical molecular dynamics (MD) simulations were performed to sample the configurational space. MD simulations were performed for 1 M NaCl and MgCl_2_, and 2 M AlCl_3_ solutions to match the experimental conditions. The classical non-polarizable force fields were employed to generate large-scale structural snapshots for subsequent QM/MMPol calculations. The details of the classical MD simulations are summarized in the ESI.†

#### *Ab initio* calculations

For the *ab initio* calculations, the systems were divided into relatively small quantum (QM) and extensive molecular mechanics (MM) parts. In this work, we employed the QM/MMPol embedding scheme^33,34^ with the AMOEBA polarizable force field.^35,36^ In the polarizable force fields, each atom is described by a static point charge and an isotropic polarizability; more sophisticated force fields such as AMOEBA also contain higher multipoles. Recently, Lipparini *et al.*^37,38^ have introduced an efficient implementation for polarizable QM/MMPol based on the Fast Multipole Method,^39^ which makes the calculation of even very large systems affordable. In our case, the MMPol part was a 20 Å-radius sphere of the respective solution surrounding the QM part. All structures were taken from classical molecular dynamics calculations. The binding energies of the aluminium cation and of a chloride anion were also checked for a 30 Å-radius sphere; the calculated values were within the error bars for smaller systems, *i.e.*, the values for a 20 Å sphere can be considered converged. The present model acknowledges the granularity of the solvent around the solute, takes into account the effect of varying ionic strength of the solutions, and allows us to control the convergence of the energetics with the increasing size of the simulation box. Importantly, the model provides an optimal treatment of electronic polarization which is instrumental for a correct description of the ionization energetics.

For the QM part, we considered two models – (1) a minimal model containing a single cation and a single water molecule (or chloride anion), (2) the first solvation-shell model containing one cation and six water molecules, which corresponds to the water coordination numbers for the investigated cations.^40–46^ The considered QM/MMPol systems are shown in Fig. 1.

The core-level energies, binding energies (BEs) and energies of the lowest two-hole final states (of a triplet multiplicity) were calculated by the Maximum Overlap Method (MOM).^47^ This approach allows the variational convergence of states with specifically localized hole(s) with any ground-state method. In this work, we performed the MOM calculations at the LC-*ω* PBE level with the range-separated parameter set to a default value of 0.4 bohr^−1^ with the cc-pCVTZ basis set for a cation and the cc-PVTZ basis set for all other atoms. We did not tune the *ω* paratemer for particular systems or geometry since we mainly aim at pointing out the relative differences in spectra and energies.

Yet, the method has some limits, in clusters with a high density of energetically close-lying electronic states, the convergence of the MOM method can be poor. The BEs and energies of the final two-hole states were calculated for a set of 20 geometries selected from the classical molecular-dynamics simulations. The BEs were calculated as an energy difference of the ground and singly ionized states, the energies of the final two-hole states were evaluated relative to the ground state.

The valence photoemission spectra for cations and their first solvation shell were also calculated with a recently introduced ionization-as-an-excitation-into-a-distant-center (IEDC) approach.^23,48,49^ The method is based on modelling the ionization from a selected orbital space as an excitation into a continuum using time-dependent density functional theory (TDDFT), similarly as was previously suggested by Stanton and Gauß^50^ as well as Coriani and Koch.^51^ As DFT is in principle an exact many-body theory, we can obtain correlated orbital energies. Similar to the MOM method, the IEDC approach was performed in the QM/MMPol arrangement at the same level of theory with a sodium cation as a distant centre (placed at a distance of 1000 Å from the system). Because in the QM/MMPol model the distant centre was not solvated, we shifted the excitation energies so that the lowest-energy TDDFT transition agrees with the first ionization energy of the cluster in the MMPol embedding scheme. In this setting, the choice of a sodium cation as a distant centre is arbitrary. A 200-frame set of 20 Å spheres was cut from the classical molecular dynamics, the QM part contained one cation and six neighbouring water molecules. Excitation energies were calculated at the LC-*ω* PBE/cc-pVTZ level; the *ω* parameter was set to 0.4 bohr^−1^.

The population analysis was performed for [Al(H_2_O)_6_]^3+^ and [Mg(H_2_O)_6_]^2+^ complexes optimized at the BH&HLYP 6-31 + g* level in the polarizable continuum. The Löwdin reduced orbital population per molecular orbital was performed in ORCA 4.2.0.^52^ All other *ab initio* calculations were performed using the locally modified current development version of Gaussian 16.^53^ All classical MD simulations were performed with the GROMACS 5.1.2 package.^54,55^

## 3 Results and discussions

### Experimental manifestation of ICD

The K-shell Auger and ICD spectra investigated in this work are produced by 1s photoionization of a metal cation in the respective electrolyte. Inner-shell photoelectron spectra resulting from this primary process were measured to determine the binding energies of the respective core levels, and are shown in Fig. S3 of the ESI.† The secondary-electron spectra measured above the metal ion 1s ionization thresholds of the NaCl, MgCl_2_, and AlCl_3_ aqueous solutions are the main subject of this article. In order to maximise their intensity, photon energies near the respective K-shell thresholds were chosen to measure the spectra shown in Fig. 2. The secondary-electron spectra associated with each sample were measured with two different photon energies to distinguish peaks from different processes: The kinetic energies of the primary photoelectron peaks disperse with photon energy, whereas the kinetic energies of the Auger and ICD features remain constant. For all three ions, the KLL Auger spectra are shown in the left panels and 2s^−1^ and 2p^−1^ photoelectron peaks are seen in the right panels of Fig. 2. In the case of Mg and Na solutions, a different baseline height (resulting from scattered electrons) was found at the two photon energies probed, probably because spectra were recorded in the proximity of the respective K-shell thresholds. To enable comparison of the ICD features, spectra were scaled and shifted on the intensity scale appropriately, which leads to most or all of the apparent difference in Mg 2p and 2s intensity for the two photon energies shown in Fig. 2b. For Na^+^, the water valence-band peaks (w^−1^) are also in the selected kinetic energy range. Note that the Na^+^ 2p peak coincides in energy with one of the water valence-band peaks, 2a_1_^−1^. The peaks with constant kinetic energy are due to different 1s^−1^ core-hole decay processes, which we outline in Fig. 3.

Among the peaks with constant kinetic energy, the most intense ones for all three ions are due to the local KLL Auger decay, shown in the leftmost panels of Fig. 2. The three ions are isoelectronic with configuration 1s^2^2s^2^2p^6^, which implies that their KLL Auger spectra resulting from the 1s^−1^ → 2p^−2^ + e^−^_Aug_ decay process should be (and are) relatively similar in terms of multiplet pattern, with the two peaks in this energy range representing the ^1^S and ^1^D states of the 2p^−2^ configuration. Similar decays ending up in 2s^−2^ and 2s^−1^ 2p^−1^ states occur at lower kinetic energies, although they are not shown in Fig. 2. In addition to the main KLL Auger decay, the intermediate M 1s^−1^ state can also relax by filling the core hole by the electrons from the 2s or 2p orbitals and emitting an electron from one of the neighbouring molecules. This ICD process can be described as M 1s^−1^ → M 2s^−1^X^−1^ + e^−^_ICD_ (ICD_2s_) or M 1s^−1^ → M 2p^−1^X^−1^ + e^−^_ICD_ (ICD_2p_), with M and X designating the metal ion and a neighbouring molecule, respectively. These ICD electrons form the non-dispersing, broad, multipeak structures observed in the right-hand panels of Fig. 2 for the Mg^2+^ and Al^3+^ ions, but not for the Na^+^ ions. The energetics of the former features are further discussed in the *Binding energies and ICD energies* Section of the ESI† and compiled in Table S1 (ESI†).

### ICD takes place between the ion and water

We will start by examining the species involved in the ICD process. The structure of the observed ICD signal should reflect the states corresponding to charge delocalization between the central ion and a neighbouring molecule (either a water molecule, as shown in Fig. 3, or a chloride anion). We assessed the contribution of the chloride anion in the ICD spectra of Al^3+^ and Mg^2+^ with the help of MD simulations. In the case of Al^3+^, our simulations showed only a very limited number of [Al(H_2_O)_5_Cl]^2+^ complexes at 2 M concentration: Only 1.5% of the observed structures featured a chloride anion in the first coordination shell. The majority of these structures also contained a second chloride anion, resulting in an average coordination number for chloride in the first hydration shell of only 0.04. For Mg^2+^, the MD simulations revealed that the fraction of contact ion pairs in solution is practically zero, in agreement with previous simulations as well as available neutron scattering and X-ray diffraction data.^40,56–58^ The sodium cation forms a limited number of contact ion pairs, however, no ICD signal was observed, preventing the study of states in which the generated charge is delocalized between the central ion and the chloride anion. In the following discussion, in accord with the above, we assume that the ICD signal observed for Mg^2+^ and Al^3+^ is dominated by water molecules in the first solvation shell.

### ICD intensity depends on the metal–ligand distance

We will compare the Na^+^, Mg^2+^, and Al^3+^ ICD spectra and focus on peak intensities next. Fig. 2 shows that the ratio of ICD intensities to the Auger signal is very different for each of the cations studied. Al^3+^ exhibits the highest relative ICD intensity, for Mg^2+^ the ICD intensity is significantly smaller, and for Na^+^ the ICD peaks effectively disappear. To quantify the differences, we compare the integrated intensities of the KLL Auger peaks (final state 2p^−2^) to the ICD_2p_ peaks (final state 2p^−1^w^−1^), *i.e.*, we determine the intensity ratio of ICD to local Auger decay. We find that the intensity ratio *I*(ICD_2p_)/*I*(KLL) is ∼0% for Na^+^, ∼2.6% ± 0.5% for Mg^2+^, and ∼5.1% ± 1% for Al^3+^. The theory of ICD predicts that the intensity should be asymptotically proportional to 1/*R*^6^, with *R* being the distance between the centres. The local solvation patterns around the three metal ions can be conveniently described by MD simulations *via* radial distribution functions (see Fig. S4 in the ESI†). The peak positions for the cation–water oxygen distribution are summarized in Table 1 together with respective coordination numbers. The mean of the distance between a cation and the instantaneously closest water oxygen is also provided in Table 1 as *r*_min_. These results highlight the tighter coordination of the solvent to the metal ion in going from Na^+^ to Mg^2+^ to Al^3+^. In other words, the increasing charge of the cation correlates with a decrease of the intermolecular distance between the cation and neighboring water molecules. Accordingly, based on the MD simulations, the ICD signatures should be 1.3 times more intense for Al^3+^ than for the Mg^2+^ and 3.2 times more intense than for Na^+^. These ratios are reasonably consistent with the experimentally observed values. We note that the binding of water to a sodium cation is rather loose. Hence, although a large number of water molecules surround the Na^+^ ions, they barely contribute to the solute secondary ionization, *i.e.* ICD, signals.

**Table 1:** Structural parameters of the hydration shell for sodium, magnesium, and aluminium cations, according to MD simulations (this work). +O *R*_max_ refers to the position of the first maximum in the cation–oxygen radial distribution function, +O *R*_min_ is the position of the first minimum in that function, +O*n*_+ O_ is the coordination number of water molecules, +O *r*_min_ is the mean distance between the cation and the closest water molecule and ±*R*_max_ is the position of the maximum in the radial distribution function between the respective cation and a chloride anion. All distances are in Å. ±*R*_max_ for Mg^2+^ is not given because this species does not form ion pairs

	Na^+^	Mg^2+^	Al^3+^
+O *R*_max_	2.28	2.04	1.86
+O *R*_min_	3.12	2.78	2.16
+O *n*_+ O_	5.58	6.00	6.00
+O *r*_min_	2.21	1.90	1.82
±*R*_max_	2.86	—	2.30

Having discussed how the change in ion–water distance from Na^+^ to Al^3+^ affects the total ICD intensity, we will now proceed to discuss a second observation related to the intensity, namely that the ICD_2p_ feature has about four times higher intensity than ICD_2s_ for both Mg^2+^ and Al^3+^. The two processes can be schematically written as 1s^−1^→2s^−1^w^−1^+ e^−^_ICD_ (ICD_2s_) and 1s^−1^→2p^−1^w^−1^+ e^−^_ICD_ (ICD_2p_). On a qualitative level, there are two reasons for this intensity difference. First, the 2s and 2p orbitals have different numbers of electrons, six in 2p and two in 2s. The number of ICD channels involving 2p is, therefore, higher than that of 2s. Second, as we mentioned before, the ICD probability scales as 1/*R*^6^ and the 2s and 2p orbitals have different spatial extents which is then reflected in relative ICD probability. Moreover, different directionality of the 2s and 2p orbitals also plays a role, for example, directional 2p orbitals might be oriented more favourably for ICD than the spherically symmetric 2s orbital.

### The ICD process takes tens of femtoseconds

The observed *I*(ICD_2p_)/*I*(KLL) intensity ratios depend on the relative branching ratios, and thus the relative timescales of the different decay channels. The 1s^−1^ state lifetime *t*_1s_ is ∼ 2.3 fs for Na^+^, ∼2.0 fs for Mg^2+^, and ∼1.7 fs for Al^3+^,^59^ and we found the experimentally observed *I*(ICD_2p_)/*I*(KLL) intensity ratios as ∼0% for Na^+^, ∼2.6% for Mg^2+^, and ∼5.1% for Al^3+^. This allows us to use the core-hole clock, in which the core-hole lifetime is used as an internal timescale for the secondary decay processes.^60–63^ We can then estimate the timescale for ICD as *t*_1s_ divided by the *I*(ICD_2p_)/*I*(KLL) ratio, which results in ∼76 fs for Mg^2+^ and ∼34 fs for Al^3+^. The non-observation of ICD for Na^+^ supports the aforementioned weaker solute–solvent interaction and suggests that the ICD process occurs much more slowly for Na^+^ compared to Mg^2+^ and Al^3+^. Similar ICD processes following 2s ionization have been reported for Na^+^, Mg^2+^, and Al^3+^.^24^ By line-shape analysis of the photoemission peaks, timescales of the IC-decays following 2s ionization were found as 3.1, 1.5, and 0.98 fs for Na^+^, Mg^2+^, and Al^3+^, respectively. These results follow the same trend as the lifetimes in the present case of ICD following 1s ionization: The decay channel becomes more efficient with a decrease in the ion–water distance, and with increasing nuclear charge *Z*. On an absolute level, the timescales of ICD following 2s ionization, however, are all substantially shorter. We tentatively interpret it as a consequence of a Coster–Kronig-like process – one of the final state holes, 2p^−1^, is in the same shell as the primary 2s^−1^ hole which makes the decay much more rapid. Radial matrix elements tabulated for the normal Auger decay of 1s and 2s holes in the metals in question do show an order of magnitude higher rate for the 2s decay.^64^ Another contributing factor may be that the overlap between the water orbitals is larger with the 2s^−1^ hole than the 1s^−1^ hole.

### ICD peaks reveal electronic structure of neighboring molecules

**Fig. 5:**
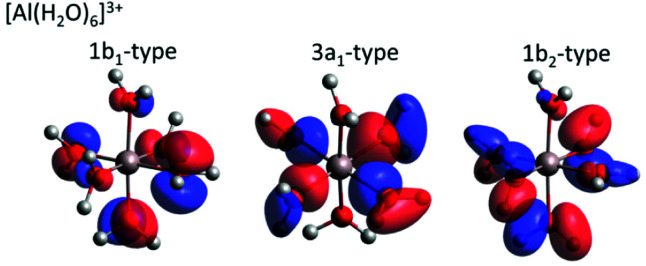
Selected molecular orbitals for the [Al(H_2_O)_6_]^3+^ complex. The Löwdin reduced orbital population per molecular orbital was performed at the BH&HLYP 6-31 + g* level in the polarizable continuum, respective molecular orbitals are depicted with an isovalue of 0.05 e.

For both Mg^2+^ and Al^3+^ ions, we can see that the ICD_2p_ and ICD_2s_ channels exhibit a substructure (see Fig. 4 containing enlarged views of the Al^3+^ and Mg^2+^ ICD peaks). The final states of the two channels are 2s^−1^w^−1^ and 2p^−1^w^−1^, respectively. The w^−1^ hole can be produced in any of the water valence orbitals, *i.e.*, 1b_1_, 3a_1_, 1b_2_, or 2a_1_. Their shape, for the [Al(H_2_O)_6_]^3+^ complex, is exemplified in Fig. 5. The figure shows how the orientation of the orbital relative to the metal ion differs between the states. The observed substructure of the ICD peaks then corresponds to localization of the final-state hole in these different orbitals. The relative positions of the peaks used for fitting the ICD structure were found practically identical to the water valence-band peaks observed in photoemission.

The observed relative intensities of the peaks associated with the different water orbitals, however, are different for ICD_2p_, ICD_2s_, and the valence photoemission spectrum of the respective salt solution. (A valence-band spectrum for NaCl solution at the photon energy of our measurements is shown in the ESI,† Fig. S1.) For both Mg^2+^ and Al^3+^, the strongest contribution in ICD_2p_ is from the water 3a_1_, followed by 1b_1_ and 1b_2_, with the 2a_1_ orbital contribution being relatively low. In ICD_2s_, however, the 1b_2_ orbital peak has a slightly higher intensity than the 3a_1_ orbital for both ions. This shows that the ICD process selects electrons from specific molecular orbitals in a way that differs from photoemission and even depends on the orbital involved in the metal ion.

A straightforward theoretical assignment of the ICD peaks would require calculations of decay rates (*e.g.*, by means of the Fano theory^65,66^). This is unfortunately intractable for the condensed phase. However, a hint about the observed shape of the ICD spectra can be provided by a simple orbital analysis; in our case, we selected the Löwdin population analysis. The preference for ICD electrons from specific molecular orbitals can conveniently be demonstrated for various molecular orbitals of water. The strongest ICD signal among the water molecular orbitals should arise from the 3a_1_-type orbitals because they are aligned along the connecting line between water oxygen and the central metal ion (typical contribution amounts to 10–11%). The percentage describes a contribution of the water molecular orbitals on a given atomic orbital of a metal atom.^67^ On the contrary, the 1b_1_-type and 1b_2_-type orbitals are oriented perpendicular to the connecting line between water oxygen and the metal ion, which is reflected in a much smaller orbital overlap (up to 2% at maximum). Reviewing the data and fits shown in Fig. 4, the orbital-overlap analysis is consistent with the observed relative intensities of the ICD_2p_ features, but the different relative intensities of the ICD_2s_ peaks show that the nature of the metal orbital also plays an important role. This calls for further research, especially as the present results indicate that ICD spectroscopy can represent a sort of ‘orbital tomography’ – in principle, it is possible to reconstruct dominant features of orbital shapes by a careful analysis of the ICD spectra following ionization of core electrons from different atoms.

It is interesting to compare this result to earlier work on core-level de-excitation processes involving several centres. A related decay mode of core-excited states is Electron Transfer Mediated Decay (ETMD), in which the energy transfer from centre to ligand, which is characteristic for ICD, is accompanied by an *electron* transfer to the centre ionized initially. This process has been studied, *e.g.*, in the de-excitation of Li^2+^ core holes in aqueous Li^+^ electrolyte solutions by some of the authors.^11,12^ Since solvated Li^+^ is devoid of any valence electrons, a 1s core hole (with a binding energy of 60.4 eV, ref. 12) can only decay when an electron is transferred from the solvation shell. Ionization by the excess energy released in this transfer has been experimentally observed, and similar to our current results showed a propensity for creating 3a_1_ vacancies in the Li solvation shell, as found by accompanying calculations.^11^ Since in that work a theoretical framework quite different from this article was used, a one-to-one comparison is not possible, but we consider it very plausible that in both cases the shape of the 3a_1_ orbital is particularly suited to create overlap with the core hole which lends efficiency to this particular decay channel. As ETMD involves electron transfer, it is clear that it can only proceed if orbital overlap is given. In ICD, orbital overlap plays a minor role: In principle, the decay is possible for two completely separated entities, rather overlap may accelerate a decay channel which is open in any case.^6,68^ Our work shows that this phenomenon can have an important impact on the shape of ICD spectra. This is reminiscent of results on the decay spectra of core holes in molecules featuring strongly electro-negative, *e.g.*, fluorine, ligands. Here a strong, ICD-like involvement of the ligands in the core-hole decay was seen^69^ and the orbital contributions of the ligands at the core-ionized centre were even found to determine the shape of the decay spectrum in some cases.^70^

### Water ligands differ from bulk water

Let us now discuss to what extent the electronic structure of the water molecules in the first solvation shell is affected by a neighbouring ion. It has been observed previously^23,71,72^ that the presence of an electrolyte has only a minor effect on the photoemission spectrum of water, even for a very high concentration of the uni–univalent electrolyte. In the valence photoemission measurements, the signal is dominated by bulk water, and the electronic structure of water molecules in the first solvation shell is very difficult to identify. In contrast, the ICD process involves practically only water molecules in the first solvation shell of the core-ionized cation. This opens up the possibility to selectively probe the electronic structure of these bound water molecules, to see how they differ from bulk water. Here, we compare the valence photoemission spectrum of bulk water and photoemission spectra of the water molecules in the first solvation shell, as simulated by the IEDC technique.

**Fig. 6:**
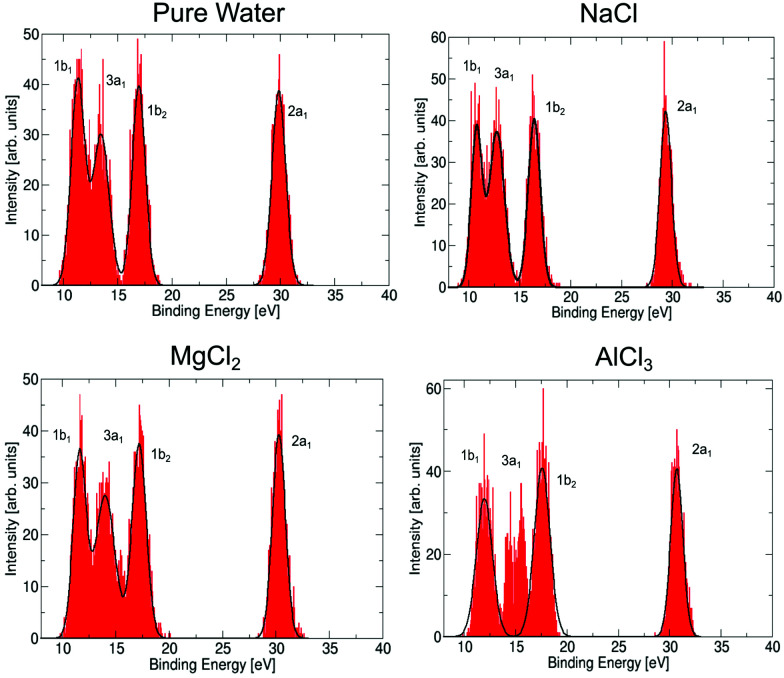
Simulated photoemission spectrum for pure water, and for water molecules directly coordinated to a metal cation in NaCl, MgCl_2_, and AlCl_3_ solutions. Spectra were calculated by the QM/MMPol IEDC approach at the LC-*ω* PBE/cc-pVTZ level.

The calculated valence photoemission spectra of pure water and water molecules directly coordinated to the cations are shown in Fig. 6, the respective peak positions are collected in Table S5 of the ESI.† It can be seen that the calculations reproduce the experimental binding energies for pure water within tenths of eV. We can observe that the cations significantly influence the binding energies of the closest water molecules. For the magnesium cation, the binding energies are shifted towards higher values (by about 0.3–0.5 eV), and the effect of the aluminium cation is even stronger, the values are shifted by about 0.6–1.3 eV. In both cases, the six water molecules forming the solvation shell are more strongly bound to the cation than to another water molecule. In the case of aluminium, the 3a_1_ peak forms a double-peak structure, the interpretation of which is unclear. Such an increase of binding energies can be expected by taking into account the electric field of a (positively charged) metal ion. This claim, however, is too simplistic, as the sodium cation exhibits the opposite effect. For Na^+^, the energies are shifted towards lower energies by 0.5–0.7 eV because Na^+^ ‘breaks’ the structure of liquid water, *e.g.*, it forms weaker bonds with water.^73^ Note that the shift in binding energies of water in the solvation shell, Δ*E*_b,*vi*_, should be taken into account in the theoretical interpretation of ICD spectra, *e.g.*, experimental values for water valence-band peaks should not be used.

### Energetics of the ICD process

We now further discuss the energetics of the ICD process, M 1s^−1^ → M 2s/2p^−1^w^−1^ + e^−^_ICD_. As can be seen in Fig. 3, the kinetic energy of the ICD electron upon 1s ionization (*E*_k_) can be expressed as the difference between the intermediate M 1s^−1^ (*E*_1s_) and M 2s^−1^/2p^−1^w^−1^ two-hole final state energies (*E*_2h_)

1 *E*_k_ = *E*_1s_ − *E*_2h_.

We can obtain the energy of the final two-hole states from the experimental data as

2 *E*_2h_ = *E*_1s_ − *E*_k_.

**Fig. 7:**
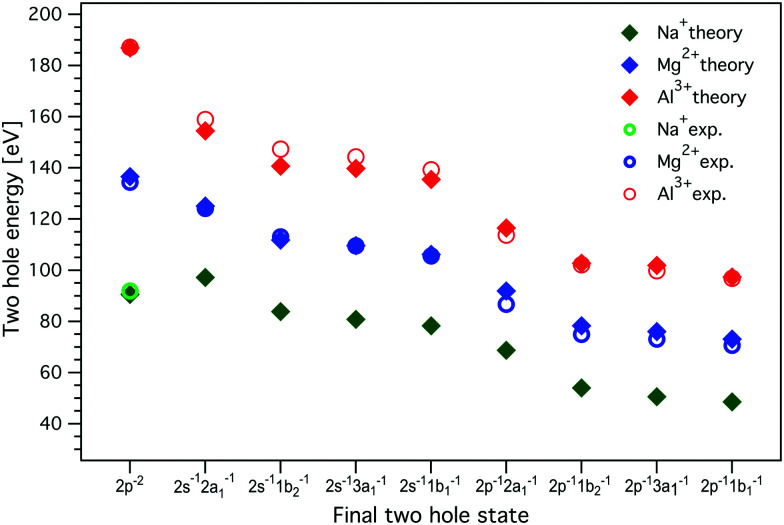
The energies of the two-hole ICD final states for Al^3+^, Mg^2+^, and Na^+^. The experimental values, measured with 1569.8 eV and 1315.25 eV photon energies for Al^3+^ and Mg^2+^, respectively, were determined from the curve fitting of the measured spectra. MOM/LC-*ω* PBE/cc-pVTZ (with cc-cPVTZ basis for the cation) energies were obtained for a minimal model containing one cation and one water molecule; the dimer was solvated by a 20 Å sphere of molecules treated at the MMPol level. The values of experimental and calculated energies are collected in Tables S2, S3, and S6 in the ESI.† Experimental errors are approximately equal to or smaller than the symbol size. No experimental values for delocalized final states are given for Na^+^, as ICD leading to the respective two-hole states could not be observed.

The resulting two-hole energies for both the Auger and ICD final states of Mg^2+^ and Al^3+^ are shown in Fig. 7 and collected in Tables S2 and S3 in the ESI.† For Mg^2+^ (Al^3+^), the experimentally obtained two-hole energies are seen to decrease from 134 (187) eV for the localized 2p^−2^ final states of the Auger decay to 70 (96) eV for 2p^−1^ 1b_1_^−1^, the lowest delocalized two-hole state reached in the ICD process. This decrease in energy is an important driving force for the ICD process, and for the localized 2p^−2^ final states this has been predicted to lead to secondary delocalization *via* the ETMD processes.^74^

To quantitatively understand this energy lowering, we can conceptually decompose the energy of the final two-hole states into three contributions

3 *E*_2h_ = *E*_b,*v*1_ + *E*_b,*v*2_ + *E*_Cp_,

where *E*_b,*vi*_ is the binding energy of the *i*^*th*^ electron participating in this process and *E*_Cp_ is the Coulomb penalty, which can be understood as the electrostatic potential energy of the positively charged metal and water ions in a close proximity. The values of *E*_b,*vi*_ and *E*_Cp_ are not directly experimentally accessible; the experiment provides bulk water valence-band peaks only. Ignoring this difference, the Coulomb penalty is generally found in the range of 3–6 eV, with a tendency towards somewhat lower values if states of s-character are involved. This is in contrast with the Coulomb penalty found for the ETMD process following the Li^+^ ionization where the Coulomb penalty was found to be very small.^11^

Theoretically, we model the energetics with the MOM approach in the minimal model containing one cation and one water molecule for Na^+^, Mg^2+^, and Al^3+^ in a polarizable embedding. In this way, we obtain the binding energies of the *i*^*th*^ electron *E*_b,*vi*_, the two-hole state energies, and the Coulomb penalty. The calculated energies are presented in Fig. 7 and collected in Tables S2 and S3 in the ESI.†

As seen in Fig. 7, the calculations reproduce the experimental values for the final two-hole energies for Mg^2+^ and Al^3+^ very well. It is important to note here that the calculated *E*_2h_ values correspond to the low-energy onset of the fitted ICD peaks (the MOM approach provides only the energies of the lowest triplet two-hole states). For Mg^2+^ and Al^3+^, the Coulomb penalty is found in the range 4–6 eV, which is reasonably consistent with the experimental estimate.

For Na^+^, there are no experimental values since we did not observe any ICD-related spectral features, but based on the good agreement between the calculation including solvation and experiment for Mg^2+^ and Al^3+^, we can use the calculated values for Na^+^. One interesting observation is that the energy difference between the localized 2p^−2^ final state to the delocalized 2p^−1^w^−1^ states is much smaller for Na^+^ than for Mg^2+^ and Al^3+^. If we regard this lowering of the two-hole state energy as a driving force for the ICD process, this may be another reason why ICD is less efficient for Na^+^ than for Mg^2+^ and Al^3+^.

As can be inferred from Table S6 in the ESI,† the theoretically predicted Coulomb penalty *E*_Cp_ is higher for Al^3+^ and Mg^2+^ than for Na^+^, which is due to the formation of highly charged pairs of Al^4+^ H_2_O^+^ and Mg^3+^H_2_O^+^ compared to Na^2+^H_2_O^+^ as the final ICD states. The energy difference between the ions is however rather minor, most probably due to a large amount of screening by the surrounding water.

### Screening effects of water

The two-hole energies for the minimal system in the gas phase are collected in Table S8 in the ESI,† respective one-electron binding energies are provided in Table S9 (ESI†). These energies exhibit the same trend for energy lowering upon delocalization as the minimal system with solvation, but the absolute two-hole energies are much higher, ≈20 eV for Na^+^, ≈40 eV for Mg^2+^, and ≈60 eV for Al^3+^. These large differences observed in the gas phase further highlight the huge screening effect of water, which minimizes the Coulomb penalty and the binding-energy shifts. The Coulomb penalty in the gas phase is a factor 1.5–2 larger than in solution; the binding-energy shifts are around 7 eV for Na^+^, 15 eV for Mg^2+^, and more than 20 eV for Al^3+^ in the gas phase compared to less than 1.3 eV in the aqueous phase. The large reduction of the two-hole energies illustrates the importance of including the solvent effects when considering the energetics of ICD.

## 4 Conclusions

X-ray photoemission spectroscopy, molecular dynamics, and *ab initio* calculations have been combined to investigate ICD channels during the decay of 1s core holes in aqueous-phase Al^3+^, Mg^2+^, and Na^+^ in chloride solutions. We observed two individual ICD features originating from the relaxation involving either the 2s or 2p levels of the cations and mostly water valence states. Importantly, each of these features displays a substructure, which has been shown to originate from the participation of different water valence orbitals. According to our calculations and the analysis of our data, transitions including the water 3a_1_ level contribute the largest share of the overall ICD signal. This is caused by the orbital overlap between the water valence states and the cation. The orientation of the water molecules toward the cation enhances the orbital overlap of the water 3a_1_ orbital, whereas the water 1b_1_ and 1b_2_ orbital overlaps are disfavoured. However, ICD channels that lead to 1b_2_ orbital emission appear to be favoured in the specific cases of Mg^2+^ and Al^3+^ ICD_2s_ processes relative to ICD_2p_, indicating that also the electronic structure of the core-hole-excited species plays a role. If chloride is present in the first solvation shell (forming a contact ion pair), we expect a significant overlap of one of the Cl^−^ 2p orbitals, too. However, according to our calculations and the experimental data, this is rarely the case. Contributions from Cl^−^ states were not discernable in the experiments.

The charge of the cation has been found to have a profound impact on the overall strength of the ICD transitions. For example, the higher the charge on the cation, the stronger the ICD signal. ICD is pronounced in the decay of 1s holes in Al^3+^ and Mg^2+^, while only Auger decay is observable in Na^+^. The enhancement of the ICD signal with the charge of the cation reflects the intermolecular distances between the cation and the water molecules in the first solvation shell, as ICD scales with 1/*R*^6^, with *R* being the distance between the interacting entities (see entries *r*_min_ in Tab. 1 for calculated, averaged values). Accordingly, the strongest ICD_2p_/Auger branching ratio could be determined for Al^3+^ with about ∼5% relative ICD efficiency. This number is about a factor of 10 higher than ICD rates, *e.g.*, in Ar clusters.^75^ A ∼5% intensity ratio also allows us to estimate the timescale of the ICD_2p_ transition to about 34 fs, based on the core-hole lifetime in Al 1s of about 1.7 fs. Note that this is about 20 times slower than the ICD decay of much shallower Al 2s holes.^14^

We have shown that the substructure of ICD spectra hinges on the electronic structure of the valence levels of the partner species during the de-excitation. One may regard the ICD spectrum as a projection of the valence states, but with the relative intensities and peak widths modified by the orbital overlap and the geometry of the reaction partners. If used under this premise, ICD can serve as a quasi-tomographic tool to explore the combined electronic structure and local geometry of aqueous solutes and their first hydration shell simultaneously.

From a radiation-chemistry perspective it is interesting to follow the further relaxation of the ICD final state. We provide a speculative outlook here: A Coulomb explosion will immediately follow the ICD in the gas phase^6^ and in aggregates of inert species.^76^ However, we suggest that the process is more complicated in the liquid phase. An ionized water molecule, H_2_O^+^, is an extremely short-lived species; in less than 50 fs, H_2_O^+^ gives up a proton to another water molecule, forming H_3_O^+^ and a hydroxyl radical.^77^ Besides, we can speculate that a charge transfer from another water molecule to the metal cation might follow;^74^ this process would give rise to another ionized water molecule and another proton transfer would follow. The overall result would then be formation of two OH radicals and two H_3_O^+^, which would later slowly diffuse.

## Data Availability

Data relevant for this study are available at DOI: 10.5281/zenodo.6372662.

## Author contributions

G. G. and O. B. conceived the project, G. G., S. M., F. T., I. W., O. B., and B. W. performed the experiments, G. G., I. U., G. Ö. and U. H. analyzed the data, E. M. and P. S. performed all the *ab initio* calculations, B. M. and F. L. adjusted and implemented MOM into the polarizable force field, G. G., E. M., P. S., O. B., and U. H. wrote the manuscript that was discussed by all authors.

## Conflicts of interest

The authors declare no competing financial interest.

## Supplementary Material

CP-024-D2CP00227B-s001
