# Supplementary material for: Probing aqueous ions with non-local Auger relaxation
Source: Phys Chem Chem Phys. 2022 Mar 23;24(15):8661–71. doi: 10.1039/d2cp00227b (PMC9007223; doi:10.1039/d2cp00227b)
Supplement: CP-024-D2CP00227B-s001 [file CP-024-D2CP00227B-s001.pdf]

Electronic Supplementary Material (ESI) for Physical Chemistry Chemical Physics.
This journal is © the Owner Societies 2022

Probing Aqueous Ions with Non-local Auger Relaxation - Electronic Supplementary Information

Geethanjali Gopakumar,*[a‡] Eva Muchová,[b‡] Isaak Unger,[a,c] Sebastian Malerz,[d] Florian Trinter,[d,e] Gunnar Öhrwall,[f] Filippo Lipparini,[g] Benedetta Mennucci,[g] Denis Céolin,[h] Carl Caleman,[a,c] Iain Wilkinson,[i] Bernd Winter,[d] Petr Slavíček,*[b] Uwe Hergenhahn,*[d] and Olle Björneholm[a]

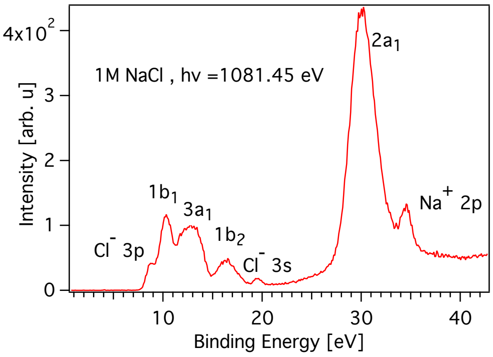

Supplementary Fig. S1 Valence band spectrum of 1 M NaCl solution measured with photon energy h$\nu = 1081.45$ eV.

Water valence band. A measurement of the water valence band recorded for a 1 M aqueous NaCl solution with h$\nu = 1081.45$ eV is shown in Fig. S1. The 3p and 3s peaks of Cl^-, and Na^+ 2p are also observed along with the water valence-band peaks. The spectrum is shown on a binding energy scale, calibrated to the literature value of the (nearly) neat water $1b_1$ peak in the absence of solute (11.33 ± 0.03 eV).[1] The structures of the Cl^- peaks and the water valence band itself are also representative for the $MgCl_2$ and $AlCl_3$ solutions.

Peak fits of ICD features. The ICD features, fitted with Voigt profiles as described in the text, for $AlCl_3$ and $MgCl_2$ solutions measurement at the higher of two photon energies used in our work are shown in Fig. S2. The water valence-band peaks contributing to the ICD features are shown by traces of different colour. The estimated energy of these peaks, the measured energy, and the difference, as well as the Coulomb penalty of these features are given in Table S3. The ICD features are independent of the photon energy, therefore, the kinetic energies of these broad peaks are similar compared to the peak energies of the corresponding lower-photon-energy measurement, shown in Fig. 4 of the main text. The values of the Coulomb penalty are also in the same range.

Photon and kinetic energy calibration. Photon energies for the Na and Mg ICD spectra were calibrated by measuring the O 1s photoelectron line with first and second order light at the chosen photon energy. For the Al ICD experiment, the O 1s second order line was outside of the kinetic energy range of our analyzer. The photon-energy calibration was therefore carried out after calibrating the kinetic energy scale of the analyzer, see below. Assuming a linear electron-kinetic-energy scale, corrected photon energies were determined as given in the main manuscript, with a correction to the nominal photon energy of $-1.51(20)$,

[a] Department of Physics and Astronomy, Uppsala University, Box 516, SE-751 20 Uppsala, Sweden. E-mail: geethanjali.gopakumar@physics.uu.se
[b] Department of Physical Chemistry, University of Chemistry and Technology, Technická 5, Prague 6, 166 28, Czech Republic. E-mail: petr.slavicek@vscht.cz
[c] Center for Free-Electron Laser Science, DESY, Notkestrasse 85, 22607 Hamburg, Germany
[d] Molecular Physics Department, Fritz-Haber-Institut der Max-Planck-Gesellschaft, Faradayweg 4-6, 14195 Berlin, Germany. E-mail: hergenhahn@fhi-berlin.mpg.de
[e] Institut für Kernphysik, Goethe-Universität Frankfurt am Main, Max-von-Laue-Strasse 1, 60438 Frankfurt am Main, Germany
[f] MAX IV Laboratory, Lund University, Box 118, SE-22100 Lund, Sweden
[g] Department of Chemistry and Industrial Chemistry, University of Pisa, Via Giuseppe Moruzzi 13, 56124 Pisa, Italy
[h] Synchrotron SOLEIL, L'Orme des Merisiers Saint-Aubin, BP 48, 91192 Gif-sur-Yvette Cedex, Paris, France
[i] Department of Locally-Sensitive & Time-Resolved Spectroscopy, Helmholtz-Zentrum Berlin für Materialien und Energie, 14109 Berlin, Germany
‡ These authors contributed equally to this work.

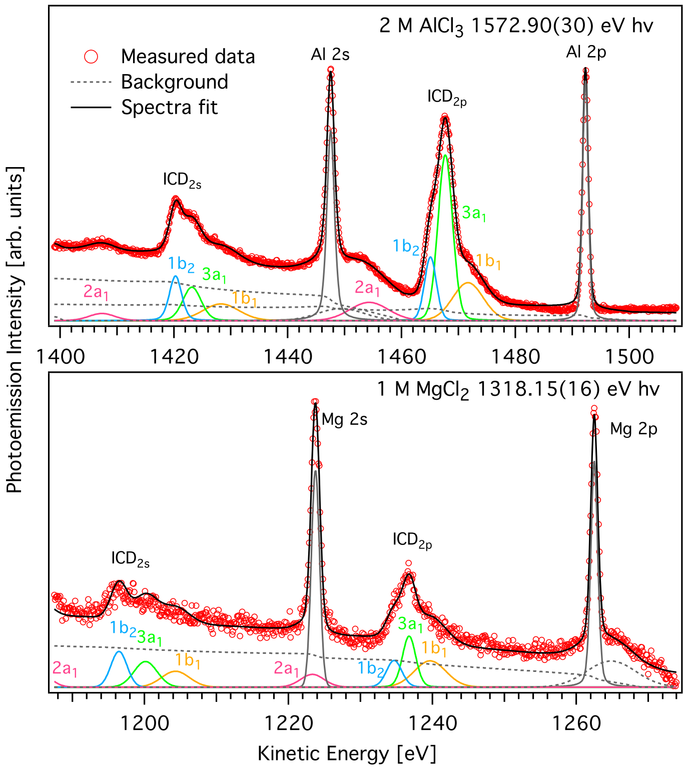

Supplementary Fig. S2 Curve fitting of ICD and photoelectron peaks of $AlCl_3$ and $MgCl_2$ spectra measured with $h\nu$ 1572.9 eV and 1318.15 eV, respectively. The Voigt profiles that are used to fit the ICD structure correspond to different water valence band orbitals, $1b_1$ (yellow), $3a_1$ (green), $1b_2$ (blue), and $2a_1$ (purple) for both ICD_{2p} and ICD_{2s}.

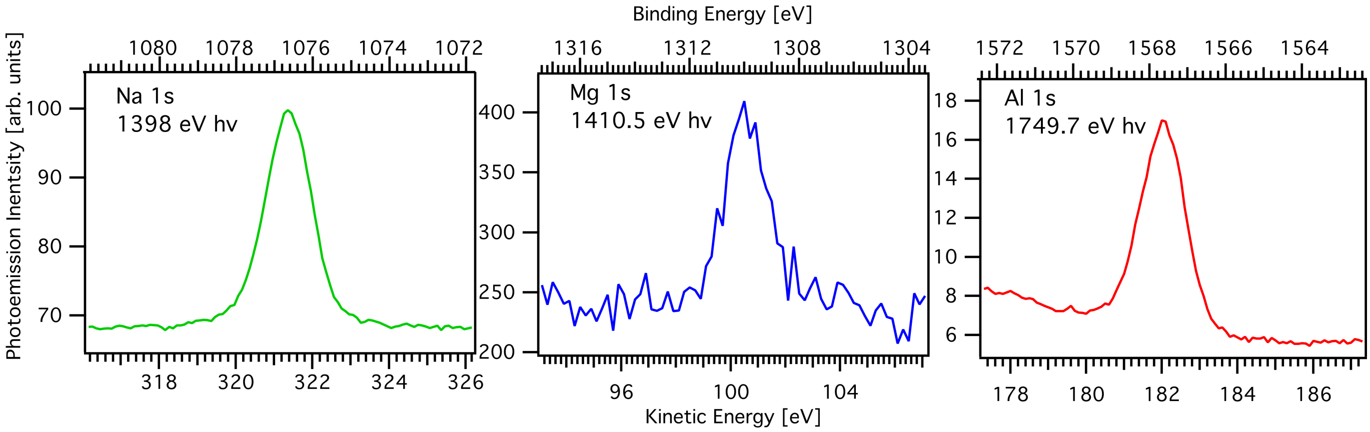

Supplementary Fig. S3 Na, Mg and Al 1s core level photoelectron spectra from aqueous 1 M NaCl, 1 M $MgCl_2$ and 2 M $AlCl_3$ solutions. The Mg 1s spectrum is from a separate calibration experiment, see text for details.

$-1.75(20)$, and $-2.1(4)$ eV being determined for the NaCl, $MgCl_2$, and $AlCl_3$-experiments, which is well in agreement with our experience from other calibration runs at the P04 beamline.

The kinetic energy scale for the Na and Mg ICD spectra was then calibrated using the corrected photon energy together with binding energies taken from Ref. 2 and collected in Table S1.

It has recently been shown that binding energies (or, more precisely, vertical ionization energies) in the liquid state can be measured on an absolute scale by referring to the cutoff formed by zero-kinetic energy electrons when a negative bias is applied to the liquid jet.[1,7] This approach, although correct, has not been used here; we instead pragmatically refer binding energies for the Na 1s, Mg 1s and Al 1s features for the solutions in question to the binding energy of the O 1s feature, setting it to its value of 538.1±0.1 eV found in neat liquid water.[1,3] By that, we are sensitive to the potential influence of a streaming potential on our

Supplementary Tab. S1 Binding energies used in this work, in eV. Values for the 1s levels were determined in this work, and are based on an O 1s binding energy for neat liquid water of 538.1 eV.[1,3] In cases of the NaCl and $AlCl_3$ solutions this value was used as a proxy for the O 1s binding energy in the electrolyte solution.[4] We averaged over the two fine-structure levels of the 2p vacancy state. Original values from Ref. 2 were given without a stated accuracy; the error shown here is our estimate considering the methodology employed in this work. Values given for the valence states of water are calculations from this work for water molecules in the first solvation shell of the respective metal center, see Tab. S5. See text for details.

Species	1s	2s	2p	3p
Na (1 M NaCl)	1076.7(4)	68.00(15)[5]	35.40(04)[5]	
Mg (1 M $MgCl_2$)	1309.9(10)	94.33(20)[2]	55.6(2)[2]	
Al (2 M $AlCl_3$)	1567.7(10)	125.15(20)[2]	80.4(2)[2]	
Cl (2 M $AlCl_3$)				9.50(15)[6]
	$1b_1$	$3a_1$	$1b_2$	$2a_1$
$H_2O(MgCl_2)$	11.62	14.01	17.21	30.29
$H_2O(AlCl_3)$	11.97	15.6	17.64	30.72

measured energies, which might be different when changing between solutions, and we neglect possible changes of the actual binding energy as a result of, *e.g.*, rearrangement of the hydrogen-bonding network. We estimate the potential error due to these shortcomings by considering the magnitude of the underlying effects: 1. Streaming potentials for highly conductive solutions were found to roughly vary between 0.1 and 0.3 eV.[8] 2. The absolute change in binding energy of water valence and solvent peaks between neat water and 2 M NaI solution was found to not exceed 0.1 eV.[7] To account for these two factors, we increase the error of binding energies determined in the course of this work by 0.2 eV.

The binding energy of the Na 1s state in 1 M aqueous NaCl solution was calibrated against the O 1s line of the same solution, measured back-to-back at equal photon energy and pass energy, giving 1076.7(4) eV independent of any photon energy and kinetic energy calibrations. A slightly higher value, but within the given error, is read from Fig. S3.

The binding energy of the Mg 1s state in 1 M aqueous $MgCl_2$ solution was determined in a separate experiment using the SOL^3PES setup at the U49/2 PGM1 beamline of BESSY II at Helmholtz-Zentrum Berlin für Materialien und Energie.[9,10] Here, firstly the photon-energy scale was calibrated by measuring the O 1s feature of the solution with first and second diffraction order light from the monochromator. Then measuring neat water (with 50 mM NaCl added), the kinetic-energy scale of the analyzer was calibrated to a value of 538.1 eV for the O 1s peak.[1] A spectrum of the Mg 1s peak recorded using the calibrated photon energy and kinetic-energy scales then yielded a binding energy of 1309.9(4) eV, where the error contains estimates for the inaccuracy of the peak-position determination, the linearity of the analyzer energy scale, the error of literature binding energy, and contributions from the streaming potential and solute-induced binding-energy changes as outlined above. The photon energy was set to 1410.5(1) eV for this measurement.

For the measurements of the Al^{3+} electrolyte, a different route had to be taken due to the photon-energy limitations of the U49/2 PGM1 beamline, resulting in it being impossible to measure the Al 1s photoemission feature in the BESSY II calibration experiments. Similarly to the Mg^{2+} experiment, the photon energy was calibrated by measuring first and second diffraction order light from the monochromator. Then the kinetic-energy scale of the analyzer was calibrated to a value of 538.1 eV for the O 1s peak.[1,3] Here, however, due to lack of time, no separate measurement of the O 1s peak could be carried out from neat liquid water. Instead we used the literature value for neat liquid water as a proxy for the O 1s peak of the 2 M $AlCl_3$ solution. A measurement of the Al KLL Auger peak (see the left hand panel of Fig. 2(a)), with the main feature being fitted by an exponentially modified Gaussian profile, then yielded a kinetic energy of 1380.9(4) eV; the error includes a 0.2 eV contribution representing potential changes of the O 1s binding energy in $AlCl_3$ solution versus water. We used this value for Al KLL to calibrate the kinetic-energy scale of our Al ICD experiment,

and subsequently determined the photon-energy correction for that experiment given above. From the Al ICD set of measurements at PETRA, a scan over the Al 1s feature at a photon energy of 1750 eV yielded a binding energy of 1567.75 eV, with the same corrections applied. This same spectrum, calibrated against the O 1s feature of $AlCl_3$ solution recorded back-to-back, yields a binding energy of 1567.63 eV, when again 538.1 eV is used as the O 1s binding energy in the solution. We summarize these two measurements as 1567.7(4) eV.

Finally, in a third experiment we tested for the consistency of the two binding-energy values determined as described above, by measuring Mg 1s of 1 M $MgCl_2$ solution and Al 1s of 2 M $AlCl_3$ solution with equal photon energy of 1760 eV (nominal value of the beamline, no correction attempted). This set of spectra yielded a binding-energy difference of the two features of 258.7 eV, while the data given above differ by 257.8 eV. This disagreement is outside of the error bars given above for the 1s measurements, albeit not by much. We point out that in this experiment Mg 1s was measured at 1760 eV photon energy, while our other experiment was at 1410.5 eV. (For Al 1s practically equal photon energies were used.) Although the binding energy should be independent of photon energy, effects like post-collision interaction in principle could contribute to the discrepancy of the two measurements we have observed. At this moment this point is speculative, however. In order to reflect our uncertainty about the true 1s binding energies, we have increased the error bars of the input values to the calculation of the expected two-hole final state energies (Fig. 7 and Tables S2 and S3) to ± 1 eV. This contribution to the systematic error manifests itself as an overall shift of all experimental Mg^{2+} or Al^{2+} energies, which is not essential for the conclusions in this paper.

For the water valence single vacancy states we found it most adequate to use the binding energies calculated in this work specifically for a water molecule directly coordinated to the respective metal center, since it it these water molecules that primarily take place in ICD of a metal vacancy. As discussed in the main text and summarized again in Tab. S5 of the Supplementary Information, these may differ notably from values found for neat liquid water.

Supplementary Tab. S2 The experimental ICD electron kinetic energies and two-hole energies. E_k (est.) is the energy estimated from the binding energies of the orbitals involved in the decay. E_k (meas.) is the experimentally measured kinetic energy. The Coulomb penalty, E_{Cp} is the difference between E_k (est.) and E_k (meas.). The two-hole energy was obtained as $E_{2h} = E_{1s} - E_k$ (meas.), and is shown in Fig. 7. E_{1s} (Mg^{2+}) = 1309.9 eV, E_{1s} (Al^{3+}) = 1567.7 eV. The associated data are collected for Al^{3+} and Mg^{2+}, measured with the lower of two hν-values used in our experiments, 1569.8 eV and 1315.25 eV, respectively. The final-state designation denotes first the vacancy in the metal center, second the vacancy in the surrounding water solvation shell. All energies are given in eV.

Final	Al^{3+} ($h\nu = 1569.8$ eV)				Mg^{2+} ($h\nu = 1315.25$ eV)			
state	E_k (est.)	E_k (meas.)	E_{Cp}	E_{2h}	E_k (est.)	E_k (meas.)	E_{Cp}	E_{2h}
$2p^{-1}$ $1b_1^{-1}$	1475.33	1470.87	4.46	96.83	1242.68	1239.21	3.47	70.69
$2p^{-1}$ $3a_1^{-1}$	1471.70	1467.88	3.82	99.82	1240.29	1236.83	3.46	73.07
$2p^{-1}$ $1b_2^{-1}$	1469.66	1465.58	4.08	102.13	1237.09	1235.01	2.08	74.89
$2p^{-1}$ $2a_1^{-1}$	1456.58	1454.00	2.58	113.70	1224.01	1223.2	0.81	86.70
$2s^{-1}$ $1b_1^{-1}$	1430.58	1428.43	2.15	139.27	1203.95	1204.34	-0.39	105.56
$2s^{-1}$ $3a_1^{-1}$	1426.95	1423.42	3.53	144.28	1202.56	1200.37	1.19	109.53
$2s^{-1}$ $1b_2^{-1}$	1424.91	1420.33	4.58	147.37	1198.36	1196.84	1.52	113.07
$2s^{-1}$ $2a_1^{-1}$	1411.83	1408.77	2.82	158.93	1185.28	1185.72	-0.44	124.18
$2p^{-2}$ Auger	1407.7	1380.9		186.8	1198.7	1175.5		134.40

The energy estimated for the ICD peaks, their measured energy, and the difference of these two numbers ('Coulomb penalty'), are given in Table S2 for the measurements shown in Fig. 4 of the main manuscript (lower photon energy), and in Table S3 for the measurements at higher photon shown in Fig. S2 of the

Supplementary Tab. S3 As Supplementary Table S2, but results from the spectra measured at $h\nu = 1572.9$ eV for Al^{3+} and 1318.15 eV for Mg^{2+}.

Final	Al^{3+} ($h\nu = 1572.9$ eV)				Mg^{2+} ($h\nu = 1318.15$ eV)			
state	E_k (est.)	E_k (meas.)	E_{Cp}	E_{2h}	E_k (est.)	E_k (meas.)	E_{Cp}	E_{2h}
$2p^{-1}\ 1b_1^{-1}$	1475.33	1471.68	3.65	96.02	1242.68	1239.89	2.79	70.01
$2p^{-1}\ 3a_1^{-1}$	1471.70	1467.69	4.01	100.01	1240.29	1236.98	3.31	72.92
$2p^{-1}\ 1b_2^{-1}$	1469.66	1465.09	4.57	102.61	1237.09	1234.9	2.19	75.00
$2p^{-1}\ 2a_1^{-1}$	1456.58	1454.31	2.27	113.39	1224.01	1223.54	0.47	86.36
$2s^{-1}\ 1b_1^{-1}$	1430.58	1428.28	2.30	139.42	1203.95	1204.52	-0.57	105.38
$2s^{-1}\ 3a_1^{-1}$	1426.95	1423.18	3.77	144.53	1202.56	1200.37	1.19	109.53
$2s^{-1}\ 1b_2^{-1}$	1424.91	1420.29	4.62	147.41	1198.36	1196.67	1.69	113.23
$2s^{-1}\ 2a_1^{-1}$	1411.83	1407.36	4.47	160.34	1185.28	1185.66	-0.38	124.25
$2p^{-2}$ Auger	1407.7	1380.9		186.8	1198.7	1175.5		134.40

Supplementary Information. See the main text for the definition of the table entries, with $E_k(\text{est.}) = E_{1s} - E_{b,v1} - E_{b,v2}$. The ICD features are independent of the photon energy, therefore, the kinetic energies of these broad peaks are similar in both measurements. The values for the Coulomb penalty are also in the same range. The columns 'E_k(est.)' and 'E_{2h}' are subject to a systematic error in the measurement of the 1s binding energy, which may lead to a common shift of all Mg or Al data points by ± 1 eV, see above. Errors for the line positions retrieved by the peak fitting are estimated as ± 0.5 eV for states involving a $3a_1$ or $1b_2$ vacancy, ± 0.8 eV for $2p^{-1}1b_1^{-1}$ states and ± 1 eV for $2s^{-1}1b_1^{-1}$ states and those involving a $2a_1$ vacancy.

Molecular dynamics. In order to generate a set of structures, we performed classical molecular simulations of NaCl, $MgCl_2$, and $AlCl_3$ solutions. These classical non-polarizable force fields allowed us to perform long molecular simulations for relatively large-scale systems. In this way, we could account for long-range polarizability in subsequent QM/MMPol calculations. The parameters for classical simulations were taken from the literature to best represent the radial distribution functions for the cation–water oxygen. We regard this parameter as crucial for subsequent calculations of the ICD states. Note, that for the magnesium cation, we used the Electronic Continuum Correction (ECC),[11,12] which employs scaled charges and a slightly modified van der Waals radius of the ions in order to best reproduce neutron scattering data specifically for the Mg–O distance in solution. Details of the classical simulations together with the Lennard-Jones parameters used in the simulations are provided in Table S4. The force field for $MgCl_2$ was taken from Ref. 13 and for NaCl from Ref. 14. The aluminium parameters were taken from Ref. 15. Water was in all cases simulated by the SPC/E model.[16] The classical simulations were performed for 1 M solutions for NaCl and $MgCl_2$ and for 2 M solution for $AlCl_3$ to match the experiment.

The simulation box for both NaCl and $MgCl_2$ contained 160 molecules of salt and an appropriate number of water molecules to match the respective density (8753 water molecules for NaCl and 8810 for $MgCl_2$). For $AlCl_3$, the simulation box contained 267 aluminium cations, 801 chloride anions, and 8472 water molecules. The total length of each simulation was 200 ns, the time step for the propagation was set to 2 fs, and 3D periodic boundary conditions were employed. The simulation temperature was set to 300 K and was controlled by a velocity-rescale thermostat with time coupling set to 0.5 ps (0.1 ps for $AlCl_3$). The pressure of the system was set to 1 bar which was controlled by the Parrinello–Rahman barostat[17] with a coupling constant of 1 ps (2 ps for $AlCl_3$). The LINCS[18] constrain algorithm of fourth order was applied to all bonds. The van der Waals interactions were truncated at 1.5 nm (1.2 nm for $AlCl_3$); the long-range electrostatic interactions were calculated by the particle mesh Ewald method.

Population analysis. The preference of ICD electron emission from specific molecular orbitals can also

atom	charge [e]	σ [nm]	ε [kJ/mol]
Na^+	1	0.2450	0.3200
Cl^-	−1	0.4400	0.4700
Mg^{2+}	1.5	0.1360	3.6610
Cl^-	−0.75	0.4100	0.4928
Al^{3+}	3	0.14472	0.906254
Cl^-	−1	0.483045	0.0534920

Supplementary Tab. S4 Parameters used in the classical molecular dynamics simulations.

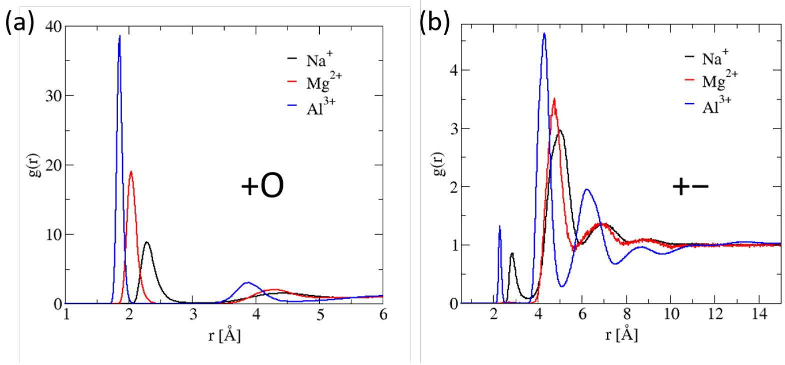

Supplementary Fig. S4 Radial distribution functions, $g(r)$, obtained from classical simulations of 1 M NaCl, 1 M $MgCl_2$, and 2 M $AlCl_3$ salt solutions at 300 K. Panels (a) and (b) show $g(r)$ as a function of distance (in Å) for cation–water (+O) and cation–anion (+−) pairs, respectively. The distances in panel (a) are measured from the cation to the water oxygen.

be conveniently demonstrated for the $[Al(H_2O)_5Cl]^{2+}$ complex. In this complex, the chloride p orbitals can be either perpendicular ($3p_x$ and $3p_y$) or parallel ($3p_z$) to the connecting line between chloride and the central cation. The orbital overlap between the chloride anion and the central ion is very different in the two cases. The Löwdin reduced orbital population per molecular orbital shows that the p_x and p_y molecular orbitals are localized only to less than 2% on the central cation while the contribution amounts to 13% for p_z. Since the ICD signal intensity should be proportional to the overlap between the orbitals of the ionized cation and of the neighbouring molecule, we can suppose that the signal arising from the $3p_z$ orbital of chloride would be quite strong. On the same basis, we can suppose that the 3s contribution would be much smaller.

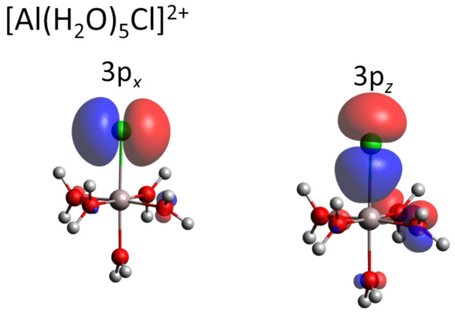

Supplementary Fig. S5 Selected molecular orbitals for the $[Al(H_2O)_5Cl]^{2+}$ complex. The Löwdin reduced orbital population per molecular orbital was performed at the BH&HLYP 6-31+g* level in the polarizable continuum, respective molecular orbitals are depicted with an isovalue of 0.05 e.

Electronic structure of water in the first solvation shell. For conciseness, we summarize here the results of our electronic structure calculations for water in the first solvation shell of a metal cation, see

Fig. 6 in the main text. Experimental binding energies for neat liquid water are shown for comparison, and were derived by using the recent absolute measurement of water's vertical ionization energy[1] for the $1b_1$ level, and the $1b_1$-$3a_1$, $1b_1$-$1b_2$ and $1b_1$-$2a_1$ energy gaps from Ref. 19 to subsequently calculate the binding energies of the other levels.

system	$1b_1$	$3a_1$	$1b_2$	$2a_1$
water (exp)[1,19]	11.33	13.67	17.51	31.07
water (theory)	11.33	13.47	16.96	29.87
$Na^+ \cdots (H_2O)_6$	10.72	12.72	16.42	29.37
$Mg^{2+} \cdots (H_2O)_6$	11.62	14.01	17.21	30.29
$[Al(H_2O)_6]^{3+}$	11.97	14.2/15.6*	17.64	30.72

Supplementary Tab. S5 Vertical ionization energy for water molecules in the neat liquid and in the first coordination shell of a metallic cation for NaCl, $MgCl_2$, and $AlCl_3$ aqueous solutions, from peak fitting to the data obtained in the QM/MMPol IEDC calculations. Energies are given in eV. The data were calculated at the LC-ωPBE/cc-pVTZ level. *This structure is a doublet peak.

Orbitals	$Na^+ \cdots H_2O$			$Mg^{2+} \cdots H_2O$			$Al^{3+} \cdots H_2O$		
	E_{2h}	E_{Cp}	E_k	E_{2h}	E_{Cp}	E_k	E_{2h}	E_{Cp}	E_k
$2p^{-1}\ 1b_1^{-1}$	48.6	3.0	1028.1	73.1	4.7	1236.8	97.4	4.4	1470.3
$2p^{-1}\ 3a_1^{-1}$	50.6	3.2	1026.1	76.0	4.8	1233.9	101.9	5.4	1465.8
$2p^{-1}\ 1b_2^{-1}$	54.0	2.9	1022.7	78.3	4.2	1231.6	102.7	4.3	1465.0
$2p^{-1}\ 2a_1^{-1}$	68.7	4.1	1008.0	91.9	4.4	1218.0	116.5	4.5	1451.2
$2s^{-1}\ 1b_1^{-1}$	78.3	4.1	998.4	106.2	4.6	1203.7	135.5	4.6	1432.2
$2s^{-1}\ 3a_1^{-1}$	80.8	4.8	995.9	109.6	5.2	1200.3	139.8	5.4	1427.9
$2s^{-1}\ 1b_2^{-1}$	83.8	4.1	992.9	111.8	4.5	1198.1	140.7	4.4	1427.0
$2s^{-1}\ 2a_1^{-1}$	97.2	4.0	979.5	125.1	4.4	1184.8	154.5	4.6	1413.2
$2p^{-2}$ Auger	90.6		986.1	136.6		1173.3	186.9		1380.8

Supplementary Tab. S6 MOM/LC-ωPBE/cc-pVTZ (with cc-cPVTZ basis for the cation) cation two-hole energies for a minimal model containing one cation and one water molecule. The dimer was solvated by a 20 Å-radius sphere of molecules, treated at the MMPol level. E_{Cp} was calculated as $E_{Cp} = E_{2h} - E_{b,v1} - E_{b,v2}$, the values of $E_{b,vi}$ are provided in Supplementary Table S7. The kinetic energy was calculated as $E_k = E_{1s} - E_{2h}$. The experimental values of E_{1s} were used since the theoretical values at the same level of theory have an error of several eV. E_{1s} (Na^+) = 1076.7 eV, E_{1s} (Mg^{2+}) = 1309.9 eV, E_{1s} (Al^{3+}) = 1567.7 eV. Two-hole states are designated like in Supplementary Table S2. All values are reported in eV.

	H_2O				cation		
	$1b_1$	$3a_1$	$1b_2$	$2a_1$	2p	2s	1s
$Na^{+}\cdots H_2O$	10.9	12.7	16.4	29.9	34.7	63.3	1073.1
exp Na^{+}					35.4[5]	68.0[5]	1076.7
$Mg^{2+}\cdots H_2O$	12.1	14.9	17.8	31.2	56.3	89.5	1307.6
exp Mg^{2+}					55.51/55.79[2]	94.33[2]	1309.9
$Al^{3+}\cdots H_2O$	12.3	15.8	17.7	31.3	80.7	118.6	1563.5
exp Al^{3+}					80.27/80.67[2]	125.15[2]	1567.7
exp H_2O[1,19]	11.33	13.67	17.51	31.07			

Supplementary Tab. S7 MOM/LC-ωPBE/cc-pVTZ (with cc-cPVTZ basis for the cation) energies for a minimal model containing one cation and one water molecule. The dimer was solvated by a 20 Å-radius sphere of molecules treated at the MMPol level. The values correspond to $E_{b,vi}$, e.g., binding energies of the ith electron involved in the ICD process. Experimental 1s values are from this work, experimental 2p and 2s values are from the references given at the respective values. Experimental values for water are from Supplementary Tab. S5. All values are reported in eV.

Calculated binding energies and ICD energies in the gas phase. In Supplementary Tables S8 and S9 we give the orbital energies and two-hole state energies for a minimal model consisting of a metal-water dimer, calculated without taking the effects of a polarizable medium (the surrounding water environment) into account.

	$Na^{+}\cdots H_2O$		$Mg^{2+}\cdots H_2O$		$Al^{3+}\cdots H_2O$	
	E_{2h}	E_{Cp}	E_{2h}	E_{Cp}	E_{2h}	E_{Cp}
$2p^{-1}\ 1b_1^{-1}$	68.0	4.0	110.0	7.8	156.7	9.4
$2p^{-1}\ 3a_1^{-1}$	73.2	6.9				
$2p^{-1}\ 1b_2^{-1}$	75.7*	5.8	115.3	7.5	161.8	9.3
$2p^{-1}\ 2a_1^{-1}$	89.4	6.3	128.9	7.7	175.8	9.5
$2s^{-1}\ 1b_1^{-1}$	98.9	6.3	143.2	7.7	194.6	9.3
$2s^{-1}\ 3a_1^{-1}$	101.7	6.8				
$2s^{-1}\ 1b_2^{-1}$	104.6	6.1	148.5	7.4	199.4	8.9
$2s^{-1}\ 2a_1^{-1}$	117.9	6.2	162.2	7.7	213.8	9.5

Supplementary Tab. S8 Two-hole energies like in Supplementary Table S6, for a minimal model containing one cation and one water molecule in the gas phase. The values of $E_{b,vi}$ are provided in Table S9. *Due to convergence issues, less than 20 calculations were performed. The SCF convergence for ICD states involving the $3a_1$ water orbital was very poor, therefore the data are missing.

	H_2O				cation		
	$1b_1$	$3a_1$	$1b_2$	$2a_1$	2p	2s	1s
$Na^{+}\cdots H_2O$	18.5	20.8	24.4	37.6	45.5	74.1	1083.8
$Mg^{2+}\cdots H_2O$	26.1	29.3	31.7	45.1	76.1	109.4	1327.3
$Al^{3+}\cdots H_2O$	35.3	39.8	40.5	54.3	112.0	150.0	1594.4

Supplementary Tab. S9 Binding energies like in Supplementary Table S7, for a minimal model containing one cation and one water molecule in the gas phase.

Notes and references

1 S. Thürmer, S. Malerz, F. Trinter, U. Hergenhahn, C. Lee, D. M. Neumark, G. Meijer, B. Winter and I. Wilkinson, *Chem. Sci.*, 2021, **12**, 10558–10582.

2 G. Öhrwall, N. Ottosson, W. Pokapanich, S. Legendre, S. Svensson and O. Björneholm, *The Journal of Physical Chemistry B*, 2010, **114**, 17057–17061.

3 B. Winter, E. F. Aziz, U. Hergenhahn, M. Faubel and I. V. Hertel, *The Journal of Chemical Physics*, 2007, **126**, 124504.

4 M. N. Pohl, E. Muchová, R. Seidel, H. Ali, Š. Sršeň, I. Wilkinson, B. Winter and P. Slavíček, *Chemical Science*, 2019, **10**, 848–865.

5 B. Winter, R. Weber, I. V. Hertel, M. Faubel, P. Jungwirth, E. C. Brown and S. E. Bradforth, *Journal of the American Chemical Society*, 2005, **127**, 7203–7214.

6 R. Seidel, B. Winter and S. E. Bradforth, *Annual Review of Physical Chemistry*, 2016, **67**, 283–305.

7 B. Credidio, M. Pugini, S. Malerz, F. Trinter, U. Hergenhahn, I. Wilkinson, S. Thürmer and B. Winter, *Phys. Chem. Chem. Phys.*, 2021, doi: 10.1039/D1CP03165A.

8 N. Kurahashi, S. Karashima, Y. Tang, T. Horio, B. Abulimiti, Y.-I. Suzuki, Y. Ogi, M. Oura and T. Suzuki, *The Journal of Chemical Physics*, 2014, **140**, 174506.

9 Helmholtz-Zentrum Berlin für Materialien und Energie, *Journal of large-scale research facilities*, 2016, **2**, A72.

10 R. Seidel, M. N. Pohl, H. Ali, B. Winter and E. F. Aziz, *Review of Scientific Instruments*, 2017, **88**, 073107.

11 I. Leontyev and A. Stuchebrukhov, *Physical Chemistry Chemical Physics*, 2011, **13**, 2613–2626.

12 M. Kohagen, P. E. Mason and P. Jungwirth, *The Journal of Physical Chemistry B*, 2014, **118**, 7902–7909.

13 E. Duboué-Dijon, P. E. Mason, H. E. Fischer and P. Jungwirth, *The Journal of Physical Chemistry B*, 2018, **122**, 3296–3306.

14 I. S. Joung and T. E. Cheatham, *The Journal of Physical Chemistry B*, 2008, **112**, 9020–9041.

15 T. M. C. Faro, G. P. Thim and M. S. Skaf, *The Journal of Chemical Physics*, 2010, **132**, 114509.

16 H. J. C. Berendsen, J. R. Grigera and T. P. Straatsma, *The Journal of Physical Chemistry*, 1987, **91**, 6269–6271.

17 M. Parrinello and A. Rahman, *Journal of Applied Physics*, 1981, **52**, 7182–7190.

18 T. Darden, D. York and L. Pedersen, *The Journal of Chemical Physics*, 1993, **98**, 10089–10092.

19 B. Winter, R. Weber, W. Widdra, M. Dittmar, M. Faubel and I. V. Hertel, *Journal of Physical Chemistry A*, 2004, **108**, 2625–2632.
